# Cyclin A2 Induces Human Adult Cardiomyocyte Cytokinesis and Elicits Cardiomyocyte Reprogramming and Dedifferentiation

**DOI:** 10.21203/rs.3.rs-6597490/v1

**Published:** 2025-05-15

**Authors:** Esmaa Bouhamida, Sangeetha Vadakke-Madathil, Prabhu Mathiyalagan, Amaresh K. Ranjan, Amir Khan, Cherrie D. Sherman, Paul E Miller, Andre Ghetti, Najah Abi-Gerges, Hina W. Chaudhry

**Affiliations:** 1 Icahn School of Medicine at Mount Sinai, New York, NY 10029, USA; 2 Benthos Prime Central, TX, USA; 3 Pharmazz Inc., Willowbrook, IL, USA, 60527; 4 AnaBios Corporation, San Diego, CA 92109, USA

## Abstract

Cyclin A2 (CCNA2), a master cell cycle regulator, is silenced in postnatal mammalian cardiomyocytes. We have previously demonstrated its ability to promote cardiac repair in small and large animals when delivered to the heart via a viral vector. However, the effect of CCNA2 gene delivery on cytokinesis in isolated cardiomyocytes from adult human hearts has not been explored. We designed a human gene therapy vector featuring a replication-deficient adenovirus encoding human CCNA2 driven by the cardiac Troponin T promoter to enable the expression of CCNA2 in freshly isolated human cardiomyocytes. Time-lapse live imaging of adult human primary cardiomyocytes from a 21-year-old male, a 41-year-old female, and a 55-year-old male demonstrated the induction of complete cytokinesis in human adult cardiomyocytes with preservation of sarcomere integrity in the resulting daughter cells with active calcium mobilization in redifferentiated cardiomyocytes. To elucidate the transcriptional mechanisms underlying this response, we conducted single-nucleus transcriptomics analysis of hearts isolated from adult transgenic mice that constitutively express CCNA2 in cardiomyocytes (CCNA2-Tg) and non-transgenic mice (nTg). This revealed a cardiomyocyte subpopulation enriched with cytokinesis, proliferative, and reprogramming genes in hearts obtained from CCNA2-Tg mice as compared to nTg mice. Ultra-deep bulk RNA sequencing of human adult and fetal hearts identified key reprogramming genes relevant to understanding the mechanisms of CCNA2-induced effects observed in our experimental models. These findings provide a promising path for the clinical development of CCNA2-based cardiac regenerative therapy.

## Graphical Abstract

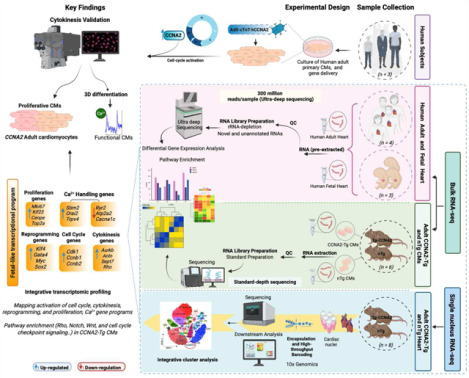

CCNA2 Induces Cytokinesis and Drives Proliferation and Reprogramming of Adult Cardiomyocytes: An Integrative Transcriptomic Analysis across Human and Mouse Models.

## Introduction

The limited proliferative capacity of adult human cardiomyocytes remains a major barrier to effective cardiac regeneration following injury, contributing to the significant morbidity and mortality associated with heart disease^1^. Scar formation via fibrosis is, therefore, the primary response to cardiac injury. Multitudes of molecular and cellular approaches have been investigated over the past 20 years aimed at regenerating the myocardium in various states of heart disease^2–4^. The use of stem/progenitor cell therapies for myocardial regeneration has demonstrated limited efficacy in clinical trials, with no conclusive evidence of actual cardiomyocyte differentiation arising from the cell types used previously in these trials. Human embryonic stem (ES) cells and induced pluripotent (iPS) cells have been evaluated in non-human primate models of heart disease with the demonstrated ability of these cell types to differentiate, at the very least, to immature cardiomyocytes with the hope that they would undergo maturation *in vivo*. However, it remains to be seen whether these approaches may be translated for human therapy^3,5^. Aside from the differentiation and generation of new cardiomyocytes, some technical concerns still need to be addressed, such as the propensity for arrhythmias noted in primate hearts treated with ES-derived and iPS-derived cardiomyocytes^5,6^.

There has been evidence of low-level cardiomyocyte turnover in the healthy human heart, but it is very limited, and this ability declines with age^7,8^. Thus, cardiac regeneration in response to injury such as myocardial infarction (MI) remains a clinical challenge. Evolution may not have favored adult mammals in this regard, but certain members of metazoan species are capable of heart regeneration, and these examples may perhaps serve to enlighten us. Urodele amphibians, such as the newt, retain a remarkable capacity to replace lost anatomical tissues through epimorphic regeneration^9^. This process relies on the local plasticity of differentiated cells near the region of injury and involves reentry to the cell cycle with loss of differentiated characteristics to generate a ‘local progenitor cell’ of restricted potentiality^9^. The adult zebrafish heart can regenerate up to 20% of its volume via a mechanism largely dependent on the proliferation of cardiomyocytes adjacent to the area of injury^10,11^. However, zebrafish harboring a temperature-sensitive mutation in *mps1*, a gene encoding a mitotic checkpoint kinase^10^, cannot regenerate the heart, and scarring and fibrosis are noted in the excised areas in a manner similar to the response of the human myocardium after injury^10,12^. In summary, the loss of a single gene can avert cardiomyocyte mitosis and mitigate the normal regenerative process, thus permitting fibrosis to proceed unimpeded.

Previously, we have reported that CCNA2 is a ‘master regulator’ of the cardiomyocyte cell cycle^13^. Unlike other cyclins, cyclin A2 complexes with its cyclin-dependent kinase partners to regulate both key transitions of the cell cycle: G1-S and G2-M^14–16^, and is silenced shortly after birth in mammalian cardiomyocytes^1,17,18^. Subsequently, we have also shown that *Ccna2* mediates cardiac repair by inducing cardiomyocyte mitoses after MI in two small animal models of MI^19–21^. As molecular mechanisms may be widely divergent across species, we performed a therapeutic efficacy study of CCNA2-mediated cardiac repair in a porcine model of MI as it closely mimics human anatomy and physiology^22^. Therapeutic delivery of *CCNA2* one week after MI in the porcine heart induced cardiomyocyte cell cycle activation *in vivo* with marked enhancement of cardiac function as noted with multimodality imaging, including magnetic resonance imaging. The experimental pigs also exhibited significantly decreased fibrosis, lack of cardiomyocyte hypertrophy, and a 55% increase in cardiomyocyte cell numbers in the peri-infarct zones. Using the same viral vector encoding CCNA2 that was delivered *in vivo*, cultured porcine adult cardiomyocytes were induced to undergo cytokinesis with preservation of sarcomere structure and captured via live imaging^22^.

For the purposes of clinical translation, we designed the next-generation gene therapy vector using a cardiomyocyte-specific promoter, cardiac Troponin T (cTnT), driving human CCNA2 expression. We then sought to determine whether this cTnT-CCNA2 vector would induce human adult cardiomyocytes to undergo cytokinesis. We employed a multiparametric approach combining live-cell imaging with high-resolution transcriptomics to uncover mechanistic insights underlying CCNA2-driven cytokinesis in adult cardiomyocytes. Live imaging microscopy enabled dynamic tracking of sarcomere integrity and cellular architecture during cytokinesis in adult human cardiomyocytes. To dissect the transcriptional programs and signaling pathways associated with *CCNA2* expression, we leveraged three complementary RNA sequencing datasets: (i) bulk RNA-sequencing of CCNA2-Tg and nTg cardiomyocytes, (ii) ultra-deep bulk RNA-sequencing of adult and fetal human hearts, and (iii) single-nucleus RNA sequencing (snRNA-seq, 10x Genomics).

This integrative approach defined the molecular framework by which CCNA2 reactivates cell cycle networks, promotes cytokinesis, and induces a regenerative transcriptional state in postnatal cardiomyocytes, offering insights for future cardiac regenerative therapies.

## Results

### cTnT-CCNA2 adenovirus vector designed for therapeutic use induces expression of CCNA2 in cultured adult human cardiomyocytes

**Figure 1.**
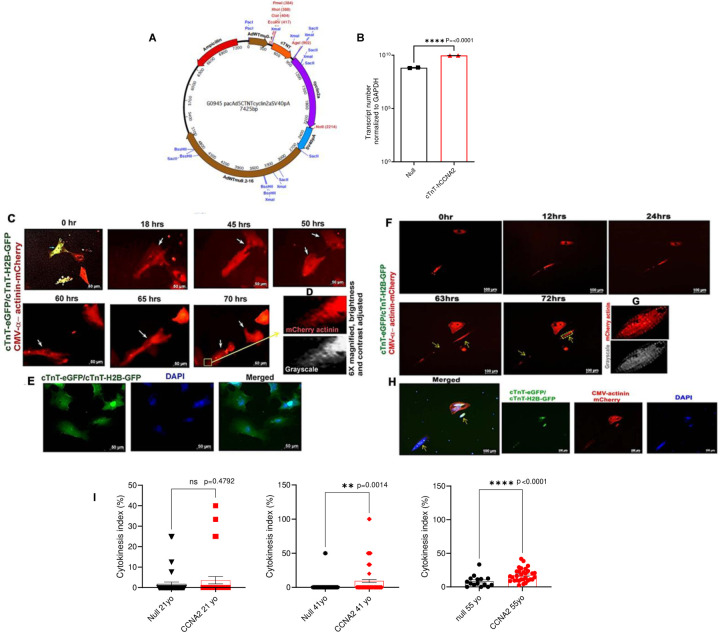
CCNA2 expression induces cytokinesis in adult human cardiomyocytes *In Vitro*. **A)** Clinical-use grade replication-deficient human adenovirus type 5 (Ad5) was used for this study. The vector was designed with cardiac Troponin T (cTnT) promoter to express human cyclin A2 (hCCNA2) specifically in cardiomyocytes. **B)** The cTnT-hCCNA2 adenovirus was used to transduce cultured adult human cardiomyocytes to induce the expression of CCNA2. Significantly higher CCNA2 expression was observed in cells transduced with cTnT-hCCNA2 compared to the control virus. **C-H)** Still images from representative time-lapse epifluorescence microscopy of cultured cardiomyocytes isolated from adult humans **C**) 55-year-old and **F)** 41-year-old. Please note that still images of 55 and 41-years-old have scale bars of 50μm and 100μm as it is shown in the movies S1 and S2 respectively. Cells were transduced with cTnT-hCCNA2, cTnT-H2B-GFP, cTnT-eGFP (to label cardiomyocytes), and CMV-α-actinin-m-Cherry adenoviruses prior to the beginning of the time-lapse imaging. **C)** At time 0 hr, a cell (yellow) expressing both cTnT (green) and α-actinin (red) was followed for 70 hrs via time-lapse microscopy. The green channel was closed, and cells were only followed through the red channel to avoid UV photo-toxicity. The observed human cardiomyocytes show the 1^st^ cell division at 50 hrs of imaging, and one of the daughter cells subsequently undergoes division at 70 hrs of imaging. **D** and **G)** At 70/72 hrs, a daughter cell is partially magnified with a grayscale version to demonstrate the presence of an intact sarcomere structure. **E, H)** 1 day after live imaging ended, these wells were fixed with subsequent labeling of nuclei with DAPI. The green fluorescence of the original cTnT-eGFP and cTnT-H2B-GFP transduction is visible, further confirming that these cells are cardiomyocytes. **I)** The cytokinetic events were counted in the control and test samples from the 21-, 41-, and 55-year-old subjects. A significantly higher rate of cytokinesis was observed in the test samples from the 41-year-old (*p*=0.001) and 55-year-old (*p*<0.0001) compared to the control. On the other hand, the control and test samples from the 21-year-old did not exhibit a significant difference in cytokinetic events (*p*=0.47). Data are represented as mean ± s.e.m.

First, we designed a therapeutic grade human CCNA2 adenovirus vector to selectively express human cyclin A2 (CCNA2) in cardiomyocytes by cloning human cDNA (NCBI Reference Sequence: NM_001237.4; 374–1672 bp) downstream to the cTnT promoter (Figure 1A). The cultured adult human cardiomyocytes were transduced with cTnT-hCCNA2 (test) versus cTnT-eGFP (control) adenoviruses with a multiplicity of infection (MOI) of 100 each for assessing the induced expression of CCNA2. We observed significantly increased expression of CCNA2 in the cultured adult human cardiomyocytes transduced with the test compared to the control adenovirus (Figure 1B). We utilized cTnT-H2B-GFP to confirm that the nuclei of the daughter cells belonged to cardiomyocytes.

### Adenoviral vector-mediated expression of CCNA2 induces cytokinesis in cultured adult human cardiomyocytes

To assess the effect of induced CCNA2 expression on cell division in adult human cardiomyocytes, we cultured adult human cardiomyocytes and studied cytokinesis *in vitro* by using live cell epifluorescence time-lapse microscopy. The adult human cardiomyocytes were plated at equal densities and transduced with cTnT-hCCNA2, cTnT-eGFP and/or cTnT-H2B-GFP and CMV-α-act-mCherry adenoviruses (test) or with cTnT-eGFP and/or cTnT-H2B-GFP and CMV-α-act-mCherry adenoviruses (control). cTnT-eGFP and/or cTnT-H2B-GFP adenovirus was transduced in both groups to confirm the initial tracking of cardiomyocytes (green) during live cell epifluorescence microscopy. For live visualization of sarcomeric structure in cardiomyocytes, cells from both groups were co-transduced with adenovirus containing α-act-mCherry, which we had constructed to allow for proper folding of the virally delivered α-actinin into the live cardiomyocyte sarcomere (CMV-α-act-mCherry) and was successfully used in cultured adult porcine cardiomyocytes^22^. This strategy enabled us to confirm cardiomyocyte identity by assessing the expression of eGFP before and after cytokinesis and tracking sarcomere dynamics during live cell imaging. This approach provides a significant advantage over antibody-based identification, which is susceptible to artifacts and restricted to a single time point after cell fixation. We observed co-expression of eGFP (green) and α-actinin (red) in cultured adult cardiomyocytes (Figure 1C and 1H; first panel). We performed time-lapse microscopic imaging of live cells to capture cardiomyocyte cytokinesis (Figure 1C, 1F, and 1H; still images from movie S1). The cytokinetic index of adult human cardiomyocytes was calculated by counting the cytokinetic events observed in 42 regions of interest (ROIs) (**Source Data** Figure 1I). The cytokinetic index was significantly higher in the test samples with cTnT-CCNA2 adenovirus transduction compared to control samples in the 41- and 55-year-olds (Figure 1I). In contrast, no significant change in the cytokinetic events between test and control samples was observed in the 21-year-old patient’s cardiomyocytes (Figure 1I). Most remarkably, sarcomere structure was preserved in the daughter cells after cytokinesis (Figure 1D and Figure 1G; upon magnification of a daughter cell, the presence of sarcomeric structure is easily noted). The daughter cells were further identified with the expression of H2B-GFP (as they were originally also transduced with cTnT-eGFP and/or H2B-GFP) and noted to be mononuclear after they had been fixed and stained with DAPI (Figure 1E and 1H). Clusters of other cardiomyocytes with expression of eGFP could be seen adjacent to the daughter cells (Figure 1E).

**Figure 2.**
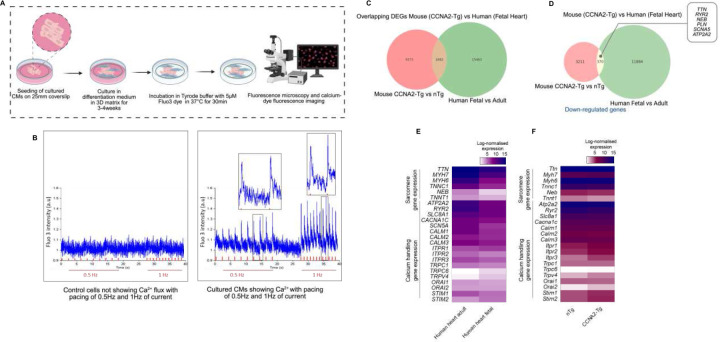
Differentiation of cultured adult human cardiomyocytes. Cultured adult human cardiomyocytes typically undergo dedifferentiation and were then subjected to 3D redifferentiation culture conditions and examined for Ca^2+^ flux. **A)** Overview of the 3D culture method of differentiation, Ca^2+^ flux measurement, and fluorescence microscopy in adult human cardiomyocytes. **B)** Representative Ca^2+^ flux graphs of re-differentiated cultured adult human cardiomyocytes. Ad5-cTnT-hCCNA2 transduced adult human cardiomyocytes show Ca^2+^ flux with pacing of 0.5 and 1 Hz of current, while no Ca^2+^ flux was detected in the non-Ad5-cTnT-hCCNA2 transduced cardiomyocytes. **C)** Venn diagram showing the overlap of differentially expressed genes between CCNA2-Tg versus non-nTg mouse cardiomyocytes and human fetal versus adult hearts. **D)** Venn diagram displaying the subset of downregulated genes shared between the CCNA2-Tg mouse hearts and the fetal human heart. These genes include several associated with cardiomyocyte maturation. **E)** Heatmap depicting the expression of Ca^2+^ handling, contractility, and sarcomere-associated genes in human adult versus fetal hearts, **F)** Comparison of gene expression in cardiomyocytes from CCNA2-Tg versus nTg mice, highlighting differences in Ca^2+^ regulatory genes and contractile machinery. Positive values indicate upregulation in adult hearts relative to fetal hearts. Data represent normalized RNA-sequencing expression values. The illustration in (**A**) was created using BioRender (https://biorender.com).

Next, we assessed the functional differentiation of cardiomyocytes by measuring the Ca^2+^ transients in CCNA2-expressing cardiomyocytes using live-cell calcium imaging under pacing conditions (0.5 Hz and 1 Hz; Figure 2A). Cardiomyocytes transduced with Ad5-cTnT-hCCNA2 exhibited active Ca^2+^ transients (Figure 2B). These findings demonstrate that CCNA2 drives cytokinesis while preserving sarcomere integrity and calcium mobilization in adult human cardiomyocytes, suggesting functional differentiation.

### CCNA2 reprogramming induces fetal gene signatures for calcium handling and sarcomere dynamics

To quantify the developmental and reprogramming landscape, we performed ultra-deep bulk RNA-sequencing of fetal (n=3) and adult (n=4) human hearts. This approach allowed us to compare the molecular signatures of CCNA2-induced cardiomyocyte reprogramming with naturally occurring fetal-like states. Our ultra-deep sequencing approach enhanced our ability to detect low-abundance transcripts, improve quantification accuracy, and provide a more comprehensive transcriptomic profile. This comparison contextualizes how CCNA2 expression modulates gene networks associated with dedifferentiation, proliferation, and calcium handling, highlighting its role in cardiac plasticity and regeneration. Notably, there were overlapping differentially expressed genes (DEGs) between adult CCNA2-Tg mouse cardiomyocytes and fetal human hearts with downregulated genes in both that were associated with cardiomyocyte maturation (Figure 2C and 2D). Furthermore, we found that calcium handling and contractility-related genes, as well as sarcomere assembly genes such as *RYR2, ATP2A2, SLC8A1, CALM1, CALM2, NEB, TTN*, and *TNNC1* were differentially upregulated in adult human hearts compared to fetal human hearts (Figure 2E).

Additionally, we performed bulk RNA-sequencing of mouse cardiomyocytes with constitutive CCNA2 expression in cardiomyocytes (CCNA-Tg mice) compared to non-Tg mice (n=6 in total). Key calcium handling genes, including *Ryr2, Atp2a2, Slc8a1*, and sarcomere assembly genes as well as *Neb, Ttn*, and *Tnnc1*, were significantly downregulated in CCNA2-Tg cardiomyocytes. In contrast, genes involved in intracellular calcium release and store-operated calcium entry, such as *Itpr1, Orai2*, and *Stim2*, were upregulated in CCNA2-Tg mouse hearts, further aligning with the fetal gene expression profile (Figure 2F and S1). This suggests that *CCNA2* may regulate sarcomere assembly genes during cell division (Figure S1), thereby facilitating cardiomyocyte proliferation^23^. Furthermore, *CCNA2* appears to influence calcium mobilization post-cytokinesis, suggesting a broader role beyond cell cycle regulation, potentially impacting cardiomyocyte maturation and function.

Live-cell imaging of CCNA2-overexpression in adult human cardiomyocytes undergoing cytokinesis revealed that daughter cells retain sarcomeric integrity post-division. Despite RNA-sequencing data showing a significant downregulation of thin filament genes (*Neb, Tnn, Tnnc1*), structural sarcomere proteins such as *Tnnt2* and *Actn2* remained stable (Figure S1), suggesting that sarcomere remodeling may occur transiently to accommodate cell division without leading to complete dedifferentiation. Interestingly, transcriptomic profiling revealed upregulation of *ZEB1* in both fetal hearts and CCNA2-Tg mouse hearts, suggesting a conserved role in cardiomyocyte plasticity (Figure S1). *SNAI1*, a gene implicated in epithelial to mesenchymal transition with roles in cancer development and progression, was not consistently upregulated. However, *ZEB1*’s expression aligns with transient mesenchymal activation, which has been implicated in neonatal heart regeneration^24^.

### Single-nucleus transcriptomic profiling reveals CCNA2-driven reprogramming in selective cardiomyocytes, supported by integrative bulk RNA-seq analysis

**Figure 3.**
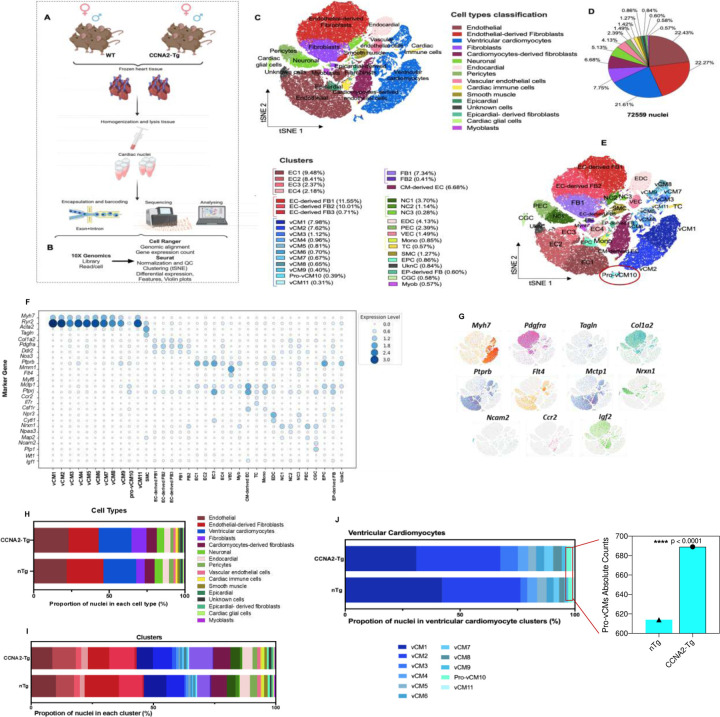
Single-nucleus RNA sequencing identified major cell types in CCNA2-Tg mice **A)** Schematic depicting the design of the snRNA-seq experiments (n=2) for each condition nTg female, CCNA2-Tg female, nTg male, and nTg male. (*n*= 8 mice in total). **B)** The analysis pipeline and single-cell barcoded library generation (10X Genomics), sequence alignment (Cell Ranger), and further analysis using R (SeuratR package). (*n*= total nuclei 72,559) **C)** t-distributed Stochastic Neighbor Embedding (t-SNE) clustering the nuclei reveals more than 16 distinct major cell types identified based on the cell-specific markers dataset provided at iRhythmics FairdomHub instance, and **D)** the pie chart showing the proportion of cells within the snRNA-seq. **E)** t-SNE of a total of 35 subclusters. **F)** Dot plot depicting the expression levels of some of the key marker genes used to classify cell types. Dot size represents the percentage of cells expressing each gene, while color intensity reflects expression levels. **G)** Feature plots highlighting the expression of selected cell-type-specific markers across the t-SNE space. **H)** Stacked bar plots representing the proportion of nuclei classified into each major cell type (top) and **I)** each identified cluster (bottom) in both CCNA2-Tg and nTg mice. **J)** Stacked bar plots displaying the proportion of nuclei in vCM clusters (left). The absolute count comparison (right) highlights a significant increase in Pro-vCM10 in CCNA2-Tg hearts relative to nTg controls (****p < 0.0001, Chi-square test), supporting the role of CCNA2 in promoting cardiomyocyte proliferation. CM: Cardiomyocytes, vCM: ventricular cardiomyocytes, EC: endothelial cells, FB: fibroblasts, EC-derived FB: endothelial-derived fibroblasts, NC: neuronal, EDC: endocardial cells. PEC: pericytes, VEC: vascular endothelial cells, Mono: monocytes, TC: T-Cells, SMC: smooth muscle cells, EPC: epicardial cells, UnkC: unknown cells, EP-derived FB: epicardial-derived fibroblasts, CGC: Cardiac glial cells, Myob: myoblasts. The illustration in (**A**) was created using BioRender (https://biorender.com).

To investigate CCNA2-induced cardiomyocyte cytokinesis in an unbiased manner, we performed snRNA-seq on hearts isolated from control non-transgenic (nTg) and *CCNA2* constitutively expressing transgenic (CCNA2-Tg) adult mice (n=8 in total). Both male and female mice aged 8–12 weeks were included. This approach enabled the identification of differentially expressed genes across cardiac clusters in both nTg and CCNA2-Tg mice in the context of CCNA2 expression. (Figure 3A, 3B, and Figure S1). Notably, snRNA-seq also captured underrepresented cell types often lost during enzymatic digestion for single cell RNA-seq, providing a comprehensive view of cardiac cellular heterogeneity^23,25^.

t-distributed Stochastic Neighbor Embedding (t-SNE) of combined nuclei profiles from both nTg and CCNA2-Tg mouse hearts revealed 16 major cardiac cell populations, including cardiomyocytes, endothelial cells, fibroblasts, cardiac, immune cells, and neuronal cells, distributed across 35 subclusters (Figure 3C–E). Cell type clusters were annotated based on expression of canonical marker genes and validated through differential expression analysis and comparison with a publicly available reference dataset (iRhythmics FairdomHub)^26,27^. (Figure 3F, 3G).

We identified several common clusters between nTg and CCNA2-Tg mice (Figure 3E, Figure S3), with differential distribution across conditions (Figure 3H and 3I). Cardiomyocyte populations were identified by the classical marker genes (*Myh7*, *Actn2*, and *Tnnc1*) in both nTg and CCNA2-Tg hearts. Cardiomyocytes that express an enhanced level of contractility genes, including the *Ryr2*, are annotated as mature cardiomyocytes^27^.

SnRNA-seq identified 11 subclusters of the ventricular cardiomyocyte (vCM) population common to both nTg and CCNA2-Tg hearts, with distinct differences in the proportion of nuclei across subclusters. Notably, the vCM1 and vCM5 were more prevalent in nTg hearts, whereas the vCM2, vCM3, vCM4, vCM6, vCM7, vCM8, vCM9, pro-vCM10, and the vCM11 were more abundant in CCNA2-Tg hearts (Figure 3J).

Subclusters vCM1, vCM2, vCM4, vCM5, and vCM11 expressed cardiomyocyte maturation-related genes, particularly sarcomere development genes, calcium (Ca^2+^) handling, and cardiac contractility genes (*Ttn*, *Pln, Ryr2*, and *Pkp2, Kcnd2*, and *Tnni3k)*, regulating cardiomyogenesis and function^28^. Notably, vCM5 also expressed non-cardiac lineage markers such as *Ebf2*, implicated in brown adipocyte differentiation^29^, and *Lsamp*, which guides neuronal connections^30^. In contrast, vCM3 and vCM7 showed increased expression of ATP synthase genes (*Atp5b* and *Atp5g3)*, while vCM4 and vCM8 co-expressed the endothelial-associated gene vCM9 was uniquely marked by *Igfbp3*, consistent with a mid-differentiation state.

Moreover, the transcriptomic profile of the Pro-vCM10 displayed a robust cell cycle signature with high expression of early-phase proliferative genes (*Top2a, Cdc20, Cdk2d, Cdk1, Prc1 Bub1, Mki67*, and *Kif23)*, and mitotic-cytokinesis drivers (*Stmn1*)^31^, alongside adhesion complex assembly and immune markers (*Cd44, and C1qa*)^32^. This transcriptional profile indicates an actively cycling, dedifferentiated cardiomyocyte population poised for regeneration. Remarkably, the proportion of Pro-vCM10 was significantly higher in CCNA2-Tg mouse hearts compared to nTg mouse hearts (Figure 3J).

**Figure 4.**
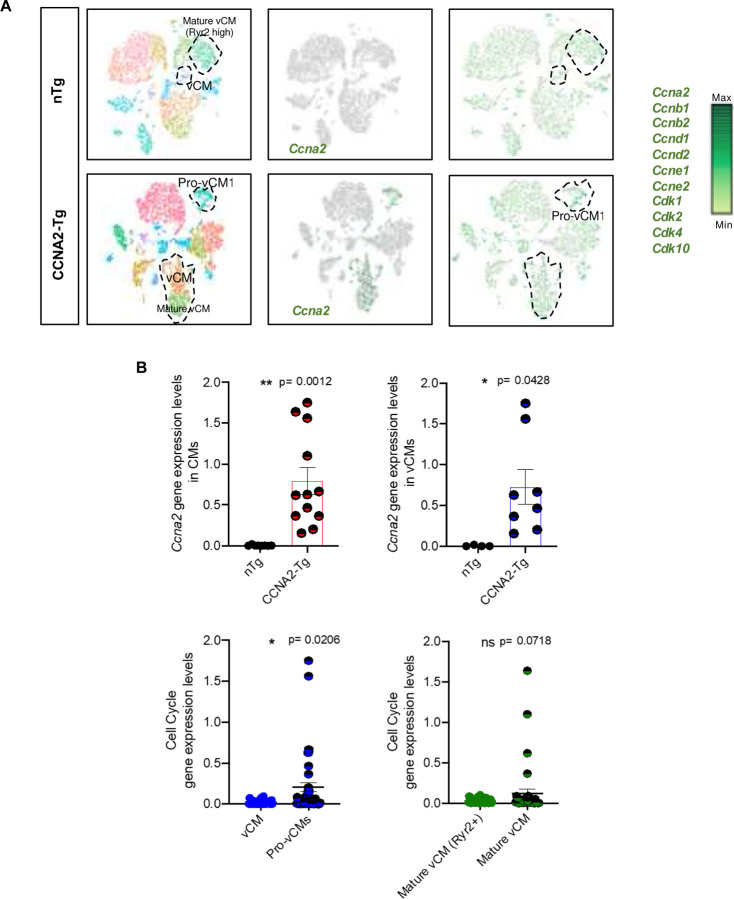
CCNA2 and cell cycle genes expression in CCNA2-Tg and nTg mice cardiomyocytes delineated by snRNA-seq. **A)** CCNA2 was expressed in the vCM, specifically in the pro-vCM subset of the cardiomyocyte population in CCNA2-Tg, and cell cycle genes projected on the t-SNE plot. **B)** Scatter plots showing the expression level of CCNA2 and cell cycle genes in cardiomyocytes of CCNA2-Tg and nTg mice. Each point represents an individual value; the mean value is represented by the horizontal line. Error bars represent s.e.m.

**Figure 5.**
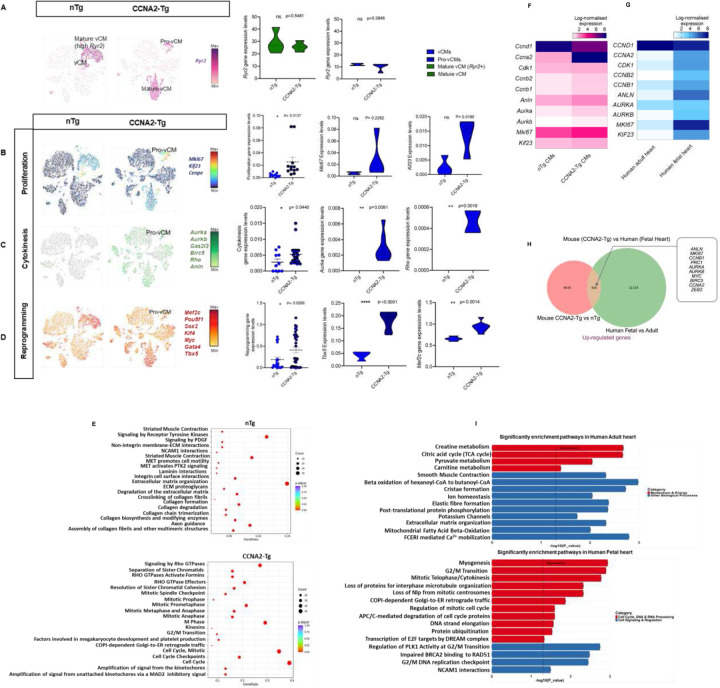
CCNA2 expression induces proliferation, cytokinesis, and reprogramming in mouse cardiomyocytes. **A)** t-SNE projection showing *Ryr2* expression across cardiomyocyte subpopulations in nTg and CCNA2-Tg mice. *Ryr2*+ cardiomyocytes correspond to a more mature ventricular cardiomyocyte (vCM) state, whereas *Ryr2* low expression cardiomyocytes represent less mature, or proliferative cardiomyocytes. Quantification of *Ryr2* expression levels among cardiomyocyte subpopulations is shown in the accompanying violin plot, highlighting differences in *Ryr2* expression across conditions. **B)** t-SNE plots and representative scatter plots and Violin plots representing the expression of key proliferation, **C)** cytokinesis, and **D)** reprogramming gene markers in both CCNA2-Tg and nTg mice. **E)** Representative dot plot of the differential gene expression analysis showing the upregulation of proliferation, cytokinesis, and reprogramming genes in CCNA2-Tg versus nTg cardiomyocytes. **F)** Heatmaps of log-transformed gene expression changes in human fetal versus adult hearts, **G)** and in CCNA2-Tg versus nTg cardiomyocytes. **H)** Venn diagram showing shared upregulated genes between CCNA2-transgenic mouse hearts and human fetal hearts. The diagram displays the overlap of upregulated genes from CCNA2-Tg versus non-nTg mouse cardiomyocytes and human fetal versus adult heart RNA-seq datasets. These shared genes are further associated with cell cycle, cytokinesis, and cardiac development, supporting a dedifferentiation and reprogramming phenotype induced by CCNA2. **I)** Pathway enrichment analysis showing significantly enriched pathways in human adult (top) and fetal (bottom) heart. In the adult heart, pathways related to metabolism and energy are enriched, based on 4,348 downregulated genes and 3,713 upregulated genes (P<0.05). In the fetal heart, cell cycle, DNA/RNA processing, and signaling pathways are enriched (P<0.05).

CCNA2 was expressed in cardiomyocytes, predominantly within the proliferative cluster identified in CCNA2-Tg hearts, which also expressed key cell cycle regulators (Figure 4A and 4B). This expression pattern remained consistent across the combined transcriptomic profiles of all subclusters (Figure S4). The pro-vCM subcluster in CCNA2-Tg mice co-expressed proliferative markers (*Mki67* and *Kif23*) and exhibited reduced *Ryr2*, consistent with a less mature phenotype (Figure 5A and 5B). Compared to nTg cardiomyocytes, where *Ryr2* expression was higher, CCNA2-Tg cardiomyocytes expressed a transcriptional signature consistent with a dedifferentiated state that may facilitate cell cycle re-entry and regeneration^33^.

Bulk RNA-sequencing further supported this profile, revealing coordinated downregulation of sarcomere, contractility genes, and upregulation of proliferative programs in CCNA2-Tg cardiomyocytes (Figure S5A and S5B), aligning with a dedifferentiated state, which facilitates cell cycle re-entry and cytokinesis. This transcriptional ‘immaturity’ may enable the proliferative potential necessary for myocardial regeneration.

To further examine CCNA2-induced cytokinesis events, we investigated whether distinct cardiomyocyte subclusters expressed genes associated with cytokinesis and/or reprogramming in CCNA2-Tg mice. Intriguingly, the pro-vCM population demonstrated upregulation of core cytokinesis regulators such as *Aurkb, Anln, Sept7*, and *Rho*, among others, with *Rho* and *Aurka* significantly elevated in CCNA2-Tg compared to nTg mice (Figure 5C). This pro-vCM subcluster also showed increased expression of reprogramming genes, including *Pou5f1, Klf4, Gata4, Myc, Sox2*, and *Tbx5*, with *Pou5f1* and *Tbx5* notably increased in CCNA2-Tg hearts compared to nTg hearts (Figure 5D). These findings were consistent with bulk RNA-sequencing analysis, which revealed increased expression of cytokinesis- and reprogramming-associated genes in CCNA2-Tg cardiomyocytes compared to nTg cardiomyocytes (Figure S5C and S5D).

Pathway enrichment analysis of CCNA2-Tg cardiomyocytes shows significant activation of cell cycle, cytokinesis, and mitotic regulation pathways compared to nTg controls. Notably, Rho GTPase signaling, spindle checkpoint regulation, and cytokinesis pathways were highly enriched (Figure 5E), indicating increased proliferation and cytoskeletal remodeling in CCNA2-Tg hearts. In contrast, nTg cardiomyocytes showed enrichment in extracellular matrix organization, collagen biosynthesis, and muscle contraction pathways, reflecting a more differentiated state. These findings indicate that CCNA2 enhances proliferation and cellular plasticity, promoting a regenerative phenotype in cardiomyocytes.

Our bulk RNA-sequencing data from mouse nTg and CCNA2-Tg cardiomyocytes yielded comparable findings. We observed upregulation of *Ccnb1* and *Ccnb2* in CCNA2-Tg cardiomyocytes compared to nTg cardiomyocytes, along with slight increases in the expression of *Cdk1* in CCNA2-Tg cardiomyocytes (Figure 5F). *Cdk1* is the sole essential cyclin-dependent kinase (*CDK*), when complexed to Cyclin A2 promotes entry into mitosis, whereas Cyclin A2/Cdk2 is critical for the G1/S transition^13,34,35^. Key cytokinesis and proliferation genes, including *Aurka, Aurkb, Anln, Mki67*, and *Kif23*, were upregulated in CCNA2-Tg cardiomyocytes, reinforcing the role of CCNA2 in cell cycle reactivation (Figure 5F). Adult human hearts exhibited downregulation of proliferative and cytokinesis-related genes (Figure 5G, 5H), whereas CCNA2-Tg cardiomyocytes retained a transcriptional profile favoring cell cycle progression and cytokinesis.

Differential expression analysis further revealed that adult human hearts exhibit a greater number of downregulated genes compared to the fetal heart (Figure 5I), consistent with widespread transcriptional silencing during cardiomyocyte maturation. In contrast, CCNA2-Tg cardiomyocytes displayed a higher number of upregulated genes relative to nTg controls (Figure S5E), indicating broad transcriptional reactivation. This inverse pattern supports the role of *CCNA2* in restoring a fetal gene expression landscape.

Pathway enrichment analysis of these differentially expressed genes confirmed upregulation of metabolism, Ca^2+^ mobilization, and ion homeostasis pathways in adult human heart, and downregulation of pathways linked to mitotic progression and developmental gene programs (Figure 5I). These findings support the hypothesis that CCNA2 reactivates a fetal-like transcriptional program in adult cardiomyocytes, promoting cell cycle re-entry without complete dedifferentiation.

Furthermore, pathway enrichment analysis revealed activation of key signaling pathways associated with cardiomyocyte plasticity, proliferation, and reprogramming in CCNA2-Tg cardiomyocytes. Specifically, the NOTCH signaling pathway and its downstream effectors (HES/HEY) were upregulated, reinforcing CCNA2’s role in progenitor-like gene activation and cardiac reprogramming. Additionally, developmental pathways such as TGF-beta regulation and Wnt interactions suggest a shift toward a more plastic, regenerative state. Increased signaling through MAPK, PDGF, and Jak-STAT pathways further supports enhanced proliferation and cytoskeletal remodeling. Furthermore, upregulation of focal adhesion, integrin interactions, and actin cytoskeleton regulation pathways indicate structural adaptations that facilitate cardiomyocyte dedifferentiation and division. Conversely, mitochondrial metabolism and oxidative phosphorylation, hallmarks of mature cardiomyocyte function, were significantly downregulated, aligning with a metabolic reversion to a fetal-like, proliferative phenotype in CCNA2-Tg cardiomyocytes. (Figure S5F, S5G). Moreover, adult CCNA2-Tg cardiomyocytes exhibit a greater number of upregulated genes compared to nTg cardiomyocytes in mice (Figure S5E).

In summary, our data highlight roles for *CCNA2* to enhance reprogramming and dedifferentiation, which ultimately elicits cardiomyocyte cytokinesis. These results provide a compelling pathway forward for the clinical development of cardiac regenerative therapy based on the manipulation of CCNA2 expression in cardiomyocytes.

## Discussion

Epimorphic regeneration is a conserved mechanism across evolutionary biology, permitting organ-specific tissue regeneration in a variety of metazoan phylogeny, including early postnatal mammals^36^. In fact, in human neonates, the ventricular myocardium responds to the pressure overload of congenital aortic stenosis through hyperplastic growth, yet the degenerative, calcific aortic stenosis of older patients elicits hypertrophic growth of the myocardium^37–39^. These clinical observations imply that the cell cycle repertoire of human neonatal cardiomyocytes is also intact, as cardiac growth is able to occur in a similar fashion to the embryonic heart. Moreover, the adult human heart is also capable of a very low rate of turnover^8^, with diminishing rates of turnover past childhood and adolescence, with cardiomyocyte cytokinesis markers not found past age 20 Strategies to induce cardiomyocyte cytokinesis in adult human hearts are of critical need in order to face the growing public health crisis of congestive heart failure. To our knowledge, most cell therapy clinical trials in heart disease have provided inconsistent evidence of improvements in left ventricular ejection fraction, with a lack of evidence that the cell types utilized actually differentiate into functional cardiomyocytes. To this end, we previously explored the effects of cell cycle manipulation in small and large animal models and successfully induced cardiomyocyte proliferation *in vivo* with significant enhancement of cardiac function in animal models^19,22^. In any translational plan to use cell cycle regulators for clinical use, two angles must be considered. Firstly, the wrong cell cycle regulators can induce cellular apoptosis^40–43^ or be ineffective if the G2/M checkpoint is not navigated^13,22,44^. Secondly, precautions must be taken to prevent the expression of cell cycle activators in extra cardiac tissues due to the potential for oncogenic transformation^45^. This safety concern prompted us to explore methods of tissue-specific activation of cyclin A2. One such method was to design a next-generation viral vector in which a cardiomyocyte-specific promoter, cardiac Troponin T, drives the expression of human cyclin A2 (cTnT-hCCNA2). Human cardiomyocytes isolated from 21-, 41- and 55-year-olds who had died of noncardiac causes were cultured utilizing adult mammalian cardiomyocyte culture methods developed by our laboratory^22^ and transduced with cTnT-hCCNA2. Cardiomyocytes from the 41- and 55-year-olds in the experimental wells underwent complete cytokinesis at a significantly higher frequency than the cardiomyocytes in the control wells. Based on our previous studies of rodent cardiomyocytes, we attribute the low rate of cytokinetic events we observed in the control wells to the reactivation of endogenous *CCNA2* with the prolonged culture of cardiomyocytes. This is a phenomenon we have previously observed and quantified in rodent cardiomyocytes, and likely not correlated with the low turnover seen in human hearts as measured by 14C dating [46] as cytokinesis markers were not noted at all in human hearts over the age 20 by Kühn and colleagues^7^. Similarly, the Frisén laboratory^46^ used stereological and 14C quantification techniques to measure cardiomyocyte exchange. They found that most cardiomyocytes were never exchanged and that the substantial replacement of existing cardiomyocytes was generated in the first 10 years of life, while the second decade of life focused on DNA polyploidization.

It is noteworthy to mention that in the adult murine context, approximately 85–90% of cardiomyocytes exist in a binucleated state^47^, indicating that the nuclear count does not precisely correspond to the cell count. The polyploidization of cardiomyocytes has been recognized as an impediment to heart regeneration^48^. It has been reported that binucleated cardiomyocytes re-entering the cell cycle are less likely to complete cytokinesis, thereby impeding the efficient regeneration of cardiac tissue following injury^36^. In adult mammalian hearts, the ratio of binucleated to mononucleated cardiomyocytes varies from one species to another. In rodent hearts, approximately 90% are binucleated, while in human hearts, this percentage is markedly lower, varying from 25–60%^49^. Thus, a greater percentage of mononuclear cardiomyocytes in the human heart would enable greater efficiency of CCNA2 gene therapy to induce cardiomyocyte proliferation as a repair strategy.

Our snRNA-Seq also confirms heightened cardiomyocyte plasticity of CCNA2 transgenic mice as we note changes in gene expression involved in cell cycle progression and reprogramming. Ca^2+^ signaling is a fundamental pathway in the regulation of cell division, cardiac contractility, and remodeling^50–52^. The increase in the cytokinetic index in the test samples from 41- and 55-year-olds shows the ability of *CCNA2* to stimulate intracellular Ca^2+^ mobilization. Of note, the transduced cTnT-hCCNA2 (test samples) cultured cardiomyocytes isolated from a 21-year-old male did not show a significant increase in cytokinetic index invoked by CCNA2 when compared to the control samples, which is consistent with reports of previous investigators^7,46^ that cell turnover and cell division can naturally occur at younger ages in humans.

SnRNA-seq has been used to identify transcriptomic changes occurring at the single-cell level, shedding light on the precise mapping of transcriptomic alterations in a cell-type-specific manner in response to cellular stimulation or gene delivery^53^. Employing snRNA-seq in intact tissues enables the capturing of cell-specific transcriptomics changes in a more precise manner while maintaining the integrity of tissue responses, compared to isolated *in vitro* transcriptomics analyses. Our strategy of using snRNA-seq is to identify cardiomyocyte-specific changes in an *in vivo* setting in a clinically relevant CCNA2-Tg model, which allowed us to map specific changes to a subpopulation of cardiomyocytes. This would not have been captured by analyzing the transcriptome of the bulk tissue of isolated cardiomyocytes using bulk RNA-sequencing strategies. Live-cell imaging demonstrates that CCNA2-transfected adult human cardiomyocytes successfully undergo cytokinesis, with daughter cells retaining what appear to be intact sarcomeric structures. However, transcriptomic analyses (bulk RNA-sequencing and snRNA-seq) revealed significant downregulation of sarcomere-related genes. These findings suggest a dynamic remodeling process during cytokinesis, where transcriptional downregulation of sarcomeric genes may occur transiently, while structural integrity is preserved through post-transcriptional mechanisms or protein stability. This highlights the intricate balance between cellular reprogramming and maintenance of cardiomyocyte identity during division.

Furthermore, the genes induced in the CCNA2-Tg model appear to represent a common mechanism underlying cytokinesis in both male and female mice, consistent with observations in human male and female hearts. Given that we identified a subset of cardiomyocytes that are reprogrammed into a more proliferative state, we can envision targeting this subset in future regenerative strategies. Unlike other regenerative approaches that separately target cardiomyocyte proliferation or reprogramming, our findings suggest that *CCNA2* integrates both processes, making it a unique therapeutic candidate for cardiac regeneration. Defining the transcriptomic state of cyclin A2-expressing cardiomyocytes and their proliferative potential in greater detail can enable us to recapitulate such proliferative cardiomyocytes both *in vitro* and *in vivo* to determine whether targeting these specific subpopulations holds greater therapeutic potential than targeting the entire cardiomyocyte population. The use of computational resources can aid our comprehension of these molecular mechanisms for more precision-guided regenerative strategies for human heart disease.

The ability of *CCNA2* to induce proliferation while maintaining cardiomyocyte function positions *CCNA2* gene therapy as a promising clinical approach, minimizing risks associated with exogenous cell transplantation, immune rejection, and off-target differentiation. Moreover, *CCNA2* appears to induce a transient mesenchymal-like state, as indicated by the upregulation of *Zeb1*, a key regulator of epithelial-to-mesenchymal transition (EMT). This suggests that CCNA2-Tg cardiomyocytes adopt a plastic phenotype to facilitate cell division, similar to mechanisms observed in neonatal heart regeneration. These findings highlight *CCNA2* as a novel and safe strategy for cardiac regeneration, offering a balance between proliferative potential and structural preservation. Ongoing studies by our laboratory have revealed silencing mechanisms of endogenous CCNA2, with antisense approaches being utilized to reverse cell cycle exit and reactivate transcription of endogenous CCNA2. These approaches may offer alternative or complementary strategies to gene therapy with CCNA2.

### Limitations

While the findings presented above are very promising, there are a few limitations to consider. Firstly, the use of freshly isolated human adult primary cardiomyocytes offers an ideal cellular model but presents several challenges. There is a high susceptibility to cell death in culture, which can hinder such experiments, and a fair amount of biological variability in getting the cells to adhere to the petri dishes. Adherence to the petri dishes is necessary to perform these cytokinesis experiments. Greater consistency with very rapid isolation from human cadavers and further refinements of the culture methods may overcome these challenges. Additionally, the use of adenovirus vectors is safe, and they are being utilized in other gene therapies that have been clinically tested. Immunogenicity is a concern, and therefore, there are ongoing investigations in our laboratory to explore alternative delivery strategies. Furthermore, *in vitro* models, though valuable, do not fully replicate the complexity of the *in vivo* cardiac environment. Nevertheless, this study is a valuable translational step forward from our previous preclinical report demonstrating that CCNA2 gene therapy induced a 55% increase in cardiomyocytes of the peri-infarct zones of the porcine heart^22^ with significant enhancement of cardiac function. CCNA2-induced cytokinesis of human adult cardiomyocytes provides a compelling pathway toward human clinical trials of CCNA2 gene therapy for heart repair.

## Methods

### Culture of adult human cardiomyocytes and *in vitro* cytokinesis study

Cardiomyocytes from adult human (21-year-old male, 41-year-old female, and 55-year-old male) heart tissue were isolated after enzymatic digestion at Anabios, San Diego, CA, and were shipped to our laboratory within 24 hours of isolation. Adult human cardiomyocytes were cultured according to our previously published adult porcine cardiomyocyte culture technique^22^ with slight modifications. In brief, upon arrival, cells were washed with serum-free Dulbecco’s modified Eagle’s medium (DMEM) (Gibco, USA) twice, and 10^5^ cells were seeded in 100mm untreated polystyrene plates (Corning, USA). Non-adherent cells were collected every 24 hours and centrifuged at 20xg for 2 min at room temperature. Cell pellet was washed with serum-free DMEM and seeded on new polystyrene plates in modified Cardiomyocyte Culture Media (mod CMC)^22^ formulated by adding 13% fetal bovine serum (FBS), 2.5% horse serum, 1X nonessential amino acid, 1mM sodium pyruvate, penicillin (100 U/ml), streptomycin (100 mg/ml), and fungizone (0.5 mg/ml) to DMEM/F12 (50:50). Cells were washed every day with serum-free DMEM, re-seeded in new polystyrene plates and cultured for 3 days. On day 4, they were seeded in glass-bottom 24-well tissue culture plates for 20 days, with the medium changed every 4th day. The wells with cardiomyocytes exhibiting adhesion and spreading were selected. Cells in these wells were trypsinized and counted, and 10^3^ cells per well were seeded in new glass-bottom tissue culture plates. After 2 days of culture, cells were divided into two groups (test and control) and were transduced with adenoviruses. The test group was transduced with cTnT-hCCNA2 along with cTnT-eGFP and/or cTnT-H2B-GFP and CMV-α-act-mCherry adenoviruses, while the control group was transduced with only cTnT-eGFP and/or cTnT-H2B-GFP and CMV-α-act-mCherry adenoviruses. MOI of adenoviruses was adjusted to 180 in each well of the test (with cTnT-hCCNA2; MOI 100, CMV-α-act-mCherry; MOI 40, and cTnT-eGFP or cTnT-H2B-GFP; MOI 40) and the control (cTnT-eGFP; MOI 140 and CMV-α-act-mCherry; MOI 40) group. After 48 hours of incubation, transduction was confirmed by observing the desired fluorescence in live cell imaging with Zeiss AxioVision Observer Z1 inverted microscope (Carl Zeiss). We also tested the differentiation potential (Ca^2+^ flux) of cardiomyocytes after cytokinesis *in vitro* by culturing them in an agarose-based semi-solid medium for 2 weeks. Cardiomyocytes were subjected to Ca^2+^ flux imaging with pacing of 0.5 Hz and 1 Hz current.

### Time-lapse microscopy

To capture cell division events in cardiomyocytes *in vitro*, we carried out live cell epifluorescence time-lapse microscopy as described in^22^. In brief, Zeiss AxioVision Observer Z1 (Carl Zeiss, Thornwood, NY, USA) inverted epifluorescence microscope in a humidified chamber in the presence of 5% CO_2_ at 37°C was used to carry out time-lapse microscopy. Multiple random points with cells expressing eGFP (green) and mCherry (red) were selected in the test and control groups. The positions were marked with the “position-list” tool in the AxioVision microscopy software (AxioVision Release 4.7, Carl Zeiss). After the first cycle of imaging, only the channel for Texas red was used (for detection of mCherry) to acquire images for 72 hours. The fluorescein isothiocyanate (green) channel of the microscope was kept closed during the time-lapse imaging to avoid cell death from exposure to ultraviolet rays in this channel. Images were taken at intervals of 30 min. The 10X objective was used for all time-lapse imaging. Time-lapse movies were generated after the end of each experiment and exported as MOV files. The time-lapse movies were analyzed, and cells that underwent successful cytokinesis were enumerated in each group. The percentage (%) of cytokinesis events was calculated for each position, and the graph was plotted.

### Cell fixation and nuclear staining

After time-lapse microscopy, cells in the glass-bottom plate were fixed with 4% paraformaldehyde at room temperature for 20 min and were stored at 4°C. For nuclear staining, cells were washed with 1x PBS then permeabilized with 0.5% Triton X-100 solution for 20 min at room temperature. Cells were washed three times with 1x PBS and incubated in DAPI solution (2.5 μg/ml) for 5 min. Cells were washed twice with 1x PBS, and imaging was carried out using a Zeiss AxioVision Observer Z1 inverted epifluorescence microscope.

### Real-time quantitative PCR

Quantitative PCRs were performed in cultured cardiomyocytes transduced with cTnT-hCCNA2 (test) or cTnT-eGFP (control) adenovirus with similar MOI (MOI 100). “SYBR Green quantitative PCR protocol” on the “StepOnePlus” real-time PCR system (Applied Biosystems, CA) was used. The PCR protocol consisted of one cycle at 95°C for 2 min, 39 cycles at 95°C for 15 sec + 60°C for 1 min, and then 4°C on hold. Gene expression was determined by using the formula 2^(39-ΔΔCT) with consideration of a CT value of 39 for a single transcript and with normalization to the endogenous control *GAPDH*.

### Animal

Cyclin A2 transgenic mice, adult males, and females 8–12 weeks old (n=4), were maintained in a B6CBA background [13, 19]. Non-transgenic (wild-type) littermates were used as controls (n=4).

### Collection of CCNA2-Tg mouse hearts and sample preparation for single-nucleus RNA-sequencing (snRNA-seq)

Frozen heart tissues from CCNA2-Tg- and nTg adult mice, male and female, were minced and transferred into a Dounce homogenizer containing ice-cold lysis buffer (Benthos Prime Central, TX, USA). Samples were homogenized gently, and nuclei were isolated. Nuclei were ensured to be free of clumps and debris by trypan blue staining under a microscope. Nuclei were then counted, and the concentration was adjusted. 10x library concentration, insert size, and quantification were checked using Qubit, Agilent bioanalyzer, and q-PCR, respectively. For data Processing and Quality Control of 10x Sequencing of snRNA-seq, the reads were processed with 10X Genomics CellRanger software (v.3.1.0) with the default parameters for each sample. Cell Ranger employs the STAR aligner for splicing-aware mapping of reads to the genome. It utilizes a transcript annotation GTF file to categorize the reads into exonic, intronic, and intergenic regions based on whether the reads align confidently with the genome. Only confidently mapped, non-PCR duplicates with valid barcodes and UMIs were used to generate a gene-barcode matrix. This involved various steps, including alignment to a reference, collapsing of unique molecular identifiers (UMIs), UMI counting, and initial quality control procedures. The output of this process was the generation of filtered gene expression matrices that exclusively contained cellular barcodes. Subsequently, the Seurat R package (v.3.1.1) was used for data quality control and downstream processing. The filtered_feature_bc_matrix generated by Cell Ranger was used for Seurat processing. Post-sequencing quality control was performed using Seurat. Cells were excluded from downstream processing in each sample if they met any of the following criteria: fewer than 200 genes were detected per cell, genes had non-zero counts in at most three cells, total feature counts exceeded 8000 (suggesting potential multiplets), more than 50% of the feature count was attributable to mitochondrial genes, or more than 5% of the feature count was attributable to hemoglobin-related genes, and doublets from all the clusters were filtered during QC using DoubletFinder version 2.0.3 (Figure S2). Raw read counts were log-normalized, cells were clustered, and markers for each cluster were identified by using the Seurat FindMarkers and FindAllMarker functions^54^. The cell clusters were annotated by using iRhythmics FairdomHub instance (https://fairdomhub.org/studies/739) and prior knowledge.

### Human fetal and adult hearts and RNA sequencing

Human fetal heart RNA (n=3 pooled, Cat #636532, Takara) and human adult heart RNA (n=4 pooled, Cat #636583, Takara) were used for bulk RNA sequencing with an increased read depth (Ultra-deep sequencing), generating approximately 300 million reads per sample. This high sequencing depth enabled the detection of rare transcripts, improved quantification accuracy, and enhanced the resolution of transcript isoforms, including novel and unannotated RNAs. For library preparation, we employed a ribosomal RNA (rRNA) depletion strategy instead of poly(A) enrichment to capture both polyadenylated (polyA^+^) and non-polyadenylated (polyA^−^) RNAs. While polyA^+^ selection primarily enriches for mRNAs and some lncRNAs, rRNA depletion allows for the inclusion of a broader spectrum of regulatory RNAs, including enhancer RNAs (eRNAs), small nuclear RNAs (snRNAs), and uncharacterized lncRNAs that may lack poly(A) tails. Additionally, this approach reduces transcript bias, preserves isoform diversity, and is better suited for degraded RNA samples.

By combining rRNA depletion with ultra-deep sequencing, we achieved a comprehensive and unbiased profile of the cardiac transcriptome. The sequence reads were mapped to the human genome (GRCh38.110) using STAR^55^ (version 2.7.9a). Using the mapped reads, the R package edgeR^56^ (version 3.30.3) was employed to calculate counts per million (CPM) values and false discovery rate (FDR)-adjusted p-values obtained through the Benjamini–Hochberg method (Benthos Prime Central, TX, USA).

### Pathway enrichment analysis

Pathway enrichment analysis was performed using the Reactome pathway analysis tool^57^. The differentially expressed genes among nTg and CCNA2-Tg conditions (log2FC > / < 1 and a P value < 0.05) were used for pathway analysis. Fold enrichment was calculated to indicate the gene expression observed in our list compared to the expected list. Categories with a fold enrichment greater than 1 were considered overrepresented, while those with a fold enrichment less than 1 were considered underrepresented in our experiment. Additionally, pathway enrichment analysis was conducted using BioPlanet via the EnrichR suite^58^, following a previously described approach^59^.

### Statistical analysis

Statistical analyses were carried out using the R package (version 3.30.3), GraphPad Prism (version 8.2.1, GraphPad), and Python (via Google Colab). Two-tailed unpaired or paired *t*-tests were used for comparison between groups with normally distributed data. Chi-square (χ^2^) tests for proportions were performed using Python libraries, including *Scipy*. The sample size (n) is reported for each analysis. Data are presented as means ± s.e.m unless otherwise indicated. Statistical significance was defined as P < 0.05.

### Study approval

This study was conducted in accordance with the Declaration of Helsinki. Adult human cardiomyocytes were obtained from Anabios (San Diego, CA) and were fully de-identified, and therefore are exempt from ethics approval. For the mouse study, the protocol was approved by the Institutional Animal Care and Use Committee (IACUC) of Mount Sinai Hospital, and all procedures adhered to Institutional Animal Care and Use Guidelines.

## Supplementary Material

1

Supplementary Files

This is a list of supplementary files associated with this preprint. Click to download.

- Therealtimeliveimagingmovieofhumanadultcardiomyocytes55yearoldmaleisdescribedinFigure1.avi
- Therealtimeliveimagingmovieofhumanadultcardiomyocytes41yearoldFemaleisdescribedinFigure1.mov

## Figures and Tables

**Figure 6.**
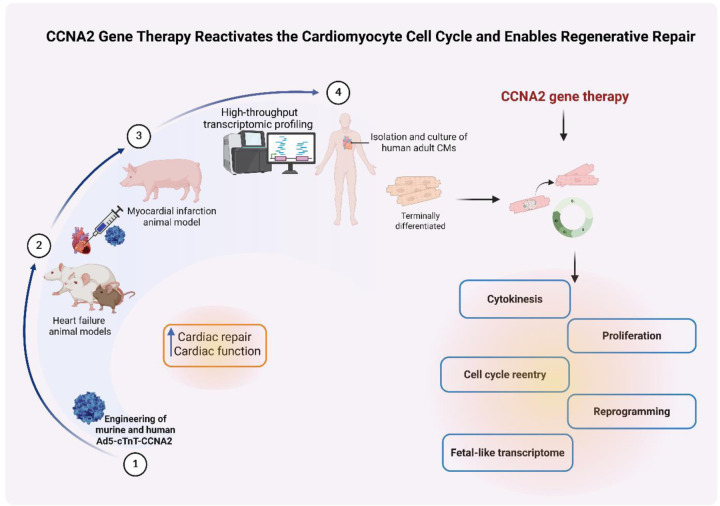
Translational roadmap and mechanistic model of CCNA2 gene therapy for cardiac regeneration. Building upon our previous *in vivo* studies in rodent and porcine myocardial infarction models, which demonstrated functional recovery via CCNA2 gene therapy (1–3) [19–22], this study provides direct evidence of complete cytokinesis in cultured human adult cardiomyocytes. Complementary transcriptomic analyses from fetal and adult human hearts, as well as CCNA2 transgenic models, reveal reactivation of fetal-like, proliferative, and reprogramming gene programs. The schematic illustrates the translational trajectory from preclinical to mechanistic insights and highlights CCNA2 as a driver of cardiomyocyte cell cycle reentry, cytokinesis, and regenerative gene expression. The illustration was created using BioRender (https://biorender.com).

## Data Availability

The processed single-nucleus data and the deep bulk RNA sequencing, including raw expression matrices and raw sequence files that support this research study’s findings, are available on the Gene Expression Omnibus GSE249433 and GSE256519, respectively.

## Abbreviations and Acronyms

Actn2: Actinin Alpha 2
Atp2a2: ATPase, SERCA2
Atp5b: ATP Synthase, Mitochondrial F1 Complex, Beta Polypeptide ATP
Atp5g3: Synthase, Mitochondrial C Subunit, Gamma Polypeptide 3
Bub1: BUB1 Mitotic Checkpoint Serine/Threonine Kinase
C1qa: Complement C1q A Chain
Ca^2+^: Calcium ion
Calm1: Calmodulin 1
Calm2: Calmodulin 2
CCNA2: Cyclin A2
CCNA2-Tg: CCNA2- transgenic mice
Cdc20: Cell Division Cycle 20
Cdk1: Cyclin-Dependent Kinase 1
Cdk2: Cyclin-Dependent Kinase 2
CGC: Cardiac glial cells
CMs: Cardiomyocytes
CPM: Counts per million
CTnT: Cardiac Troponin T
CTnT-hCCNA2: Cardiac Troponin T- human cyclin A2
E1/E2: Early region 1/Early region 2
EC: Endothelial cells
EC-derived FB: Endothelial-derived fibroblasts
EDC: Endocardial cells
Efnb2: Ephrin B2
eGFP: Enhanced Green Fluorescent Protein
EPC: Epicardial cells
EP-derived FB: Epicardial derived fibroblasts
ES: Embryonic stem cells
FB: Fibroblasts
FDR: False discovery rate
Gata4: GATA Binding Protein 4
H2B: Histone 2B
Igfbp3: Insulin-Like Growth Factor Binding Protein 3
iPS: Induced pluripotent
Kcnd2: Potassium Voltage-Gated Channel Subfamily D Member 2
Kif23: Kinesin Family Member 23
Klf4: Kruppel Like Factor 4
MI: Myocardial infarction
Mki67: Marker of Proliferation Ki-67
MOI: Multiplicity of Infection
Mono: Monocytes
Myc: MYC Proto-Oncogene, BHLH Transcription Factor
Myh7: Myosin Heavy Chain 7
Myl2: Myosin Light Chain 2
Myob: Myoblasts
NC: Neuronal cells
Neb: Nebulin
nTg: Non-transgenic mice
PEC: Pericytes
Pkp2: Plakophilin 2
Pln: Phospholamban
Pou5f1: POU Class 5 Homeobox 1, Oct4
Prc1: Protein Regulator of Cytokinesis 1
Pro-vCM: Proliferative ventricular cardiomyocyte
Ryr2: Ryanodine Receptor 2
Scn5a: Sodium Voltage-Gated Channel Alpha Subunit 5
SMC: Smooth muscle cells
SnRNA-seq: Single nucleus RNA sequencing
TC: T-Cells
Tnnc1: Troponin C1
Tnni3k: Troponin I Interacting Kinase
Top2a: Topoisomerase (DNA) II Alpha
t-SNE: T-distributed Stochastic Neighbor Embedding
Ttn: Titin
UMI: Unique molecular identifier
UnkC: Unknown cells
vCM: Ventricular cardiomyocyte
VEC: Vascular endothelial cells
α-act: Alpha-actinin
