# Supplementary material for: Cyclin A2 Induces Human Adult Cardiomyocyte Cytokinesis and Elicits Cardiomyocyte Reprogramming and Dedifferentiation
Source: Res Sq. 2025 May 15:rs.3.rs-6597490. Preprint. [Version 1] doi: 10.21203/rs.3.rs-6597490/v1 (PMC12136222; doi:10.21203/rs.3.rs-6597490/v1)
Supplement: 1 [file NIHPPrs6597490v1-supplement-1.pdf]

Supplemental Material

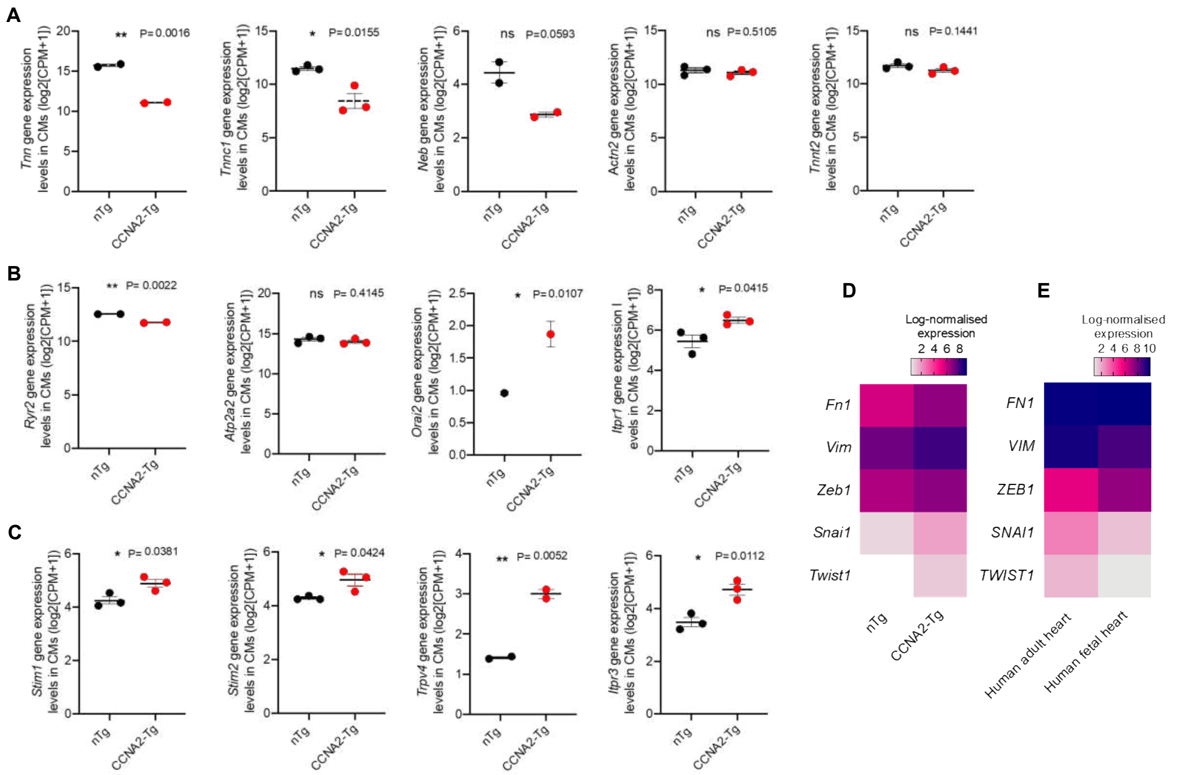

Figure S1. A) Representative scatter plots of sarcomere assembly genes *(Neb, Tnn, Tnnc1, Actn2,* and *Tnnt2),* and **B)** Ca^{2+} handling genes (*Ryr2, Atp2a2, Slc8a1*, and others) in nTg and CCNA2-Tg cardiomyocytes as delineated from bulk RNA seq. Each point represents an individual value; the mean value is represented by the horizontal line. Error bars represent SEM, with statistical significance indicated (*P*-values). ns denotes non-significant differences. **C)** Heatmap of log-normalized expression of mesenchymal transition markers (*Fn1, Vim, Zeb1, Snai1, and Twist1*) in nTg and CCNA2-Tg cardiomyocytes, as well as **D)** human adult and fetal hearts. Gene expression levels are log-transformed and normalized.

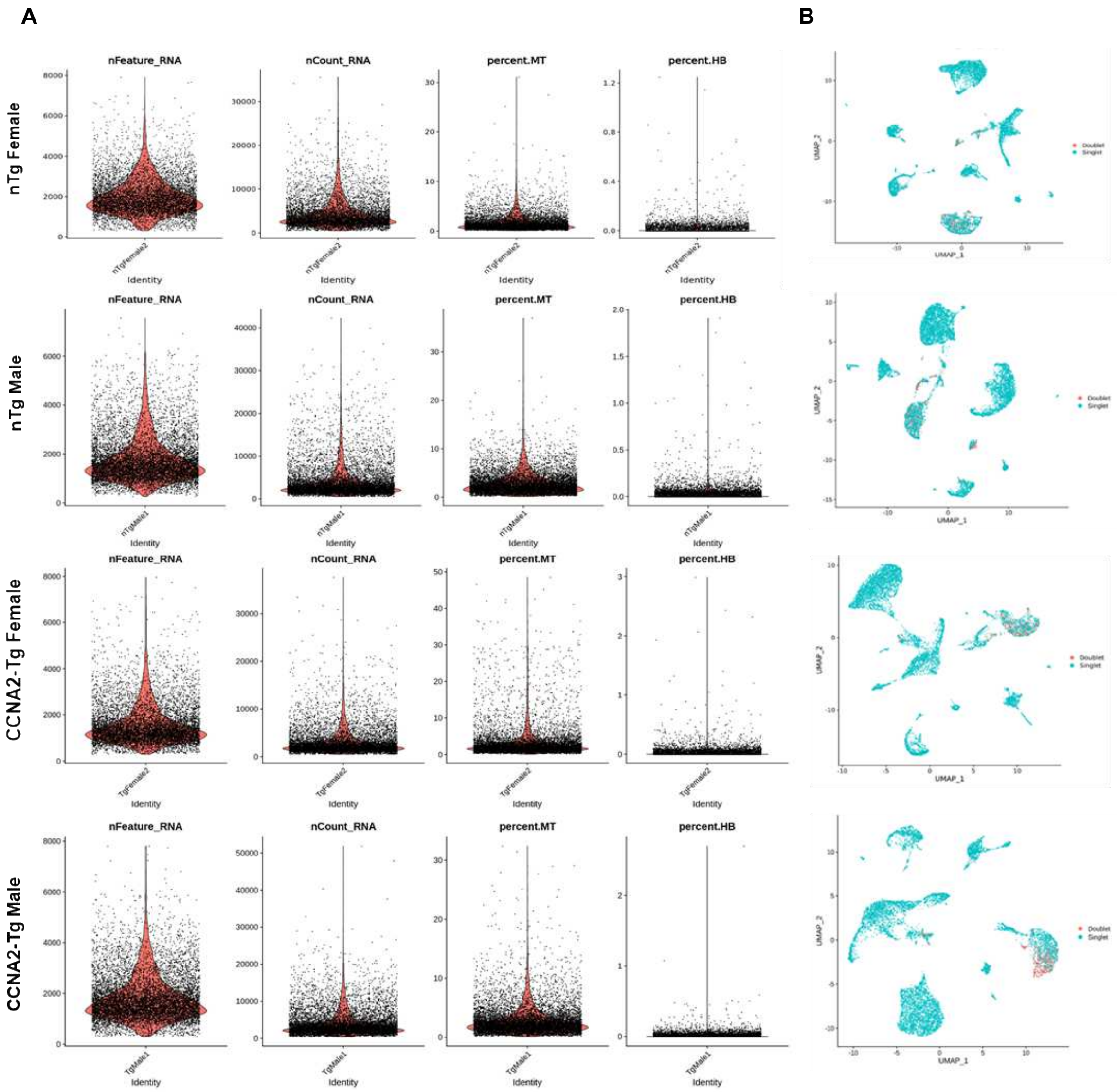

Figure S2. Quality control (QC) filtering and visualization in SnRNA-seq. A) "nFeature_RNA" indicates the count of gene features; "nCount_RNA" represents the count of UMIs; "percent.MT" signifies the proportion of counts originating from mitochondrial genes, and "percent.HB" signifies the proportion of counts stemming from Hemoglobin-related genes in all conditions nTg and CCNA2-Tg males and females. Each data point corresponds to a cell. **B)** The UMAP plots are generated from the top principal components within the clustered dataset. Each data point represents an individual cell and is color-coded based on whether it is categorized as a singlet or doublet.

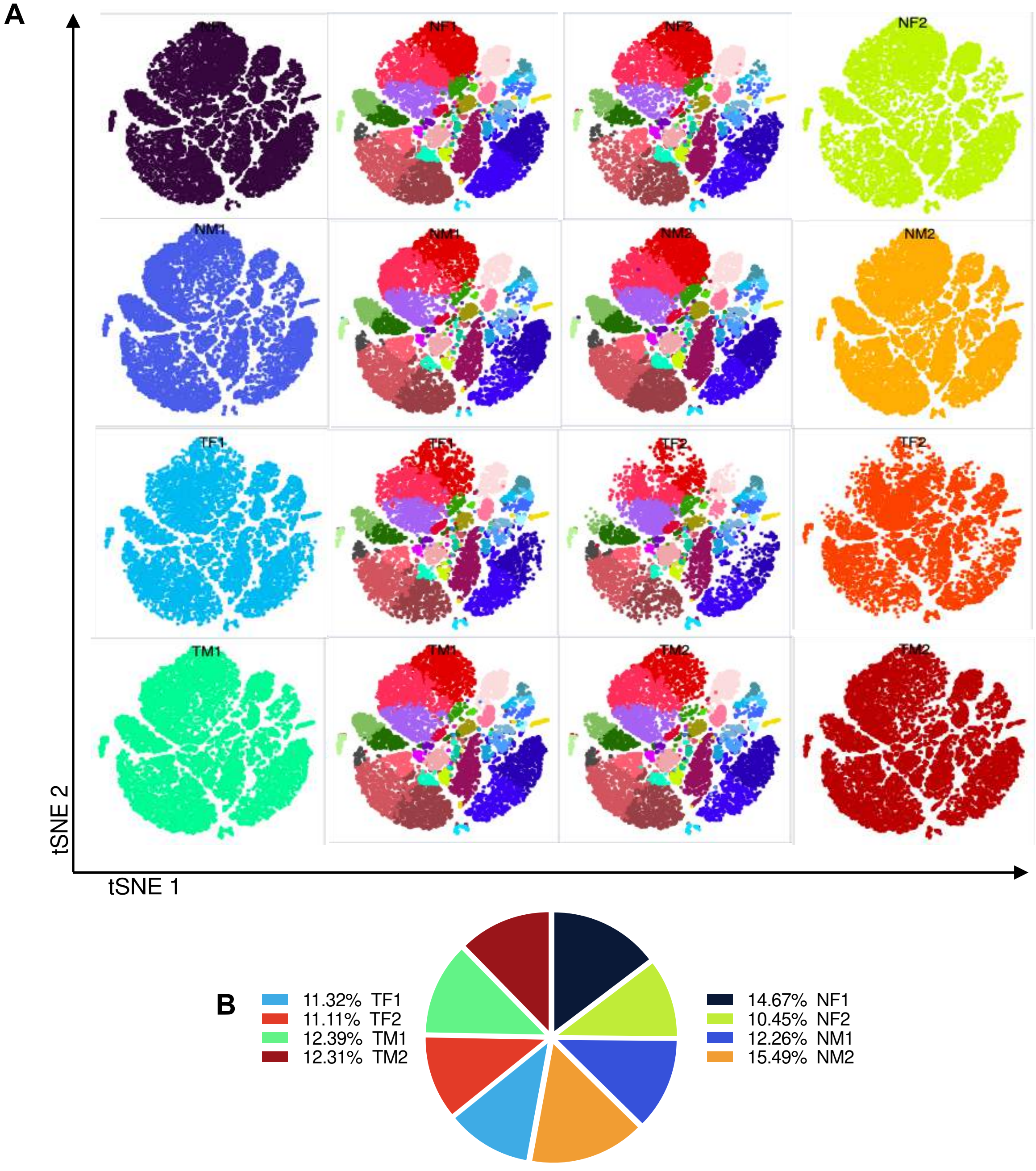

Figure S3. A) t-SNE plot clustering the nuclei of nTg and CCNA2-Tg conditions (*n*=8 total). **B)** Pie chart representing the percentage of nuclei profile in all conditions of nTg and CCNA2-Tg mice. (*n*= 72,559 total nuclei)

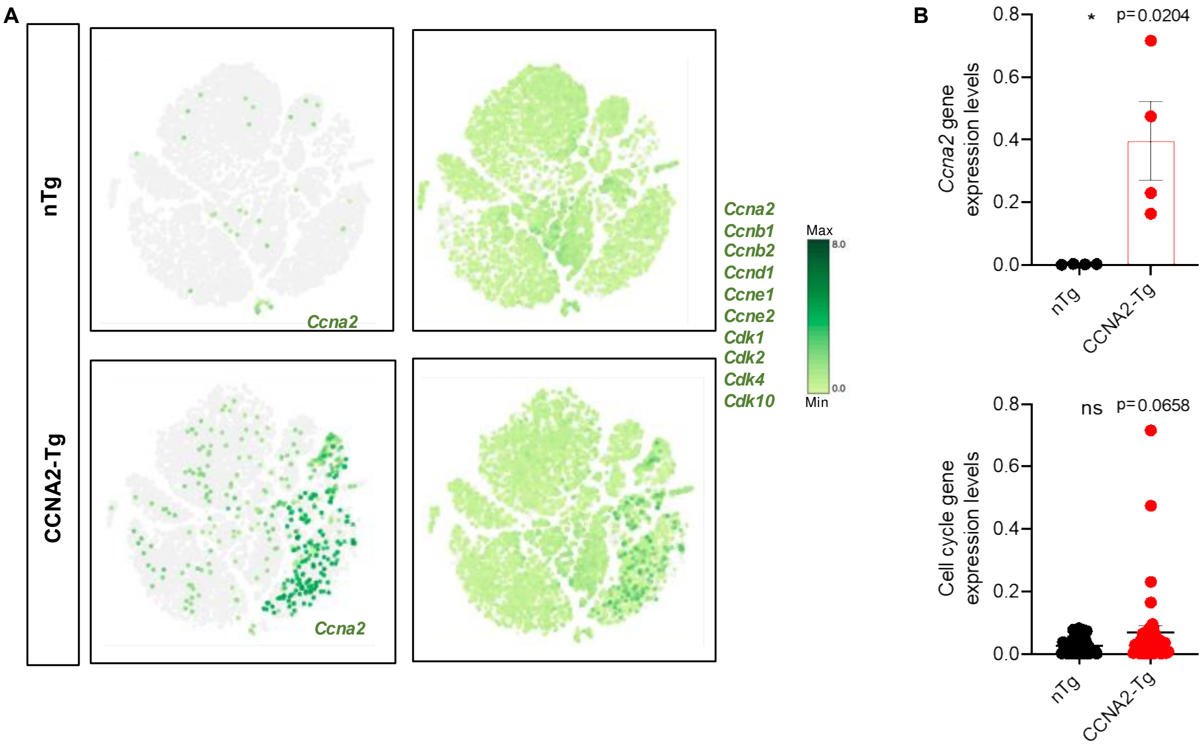

Figure S4. A) t-SNE plots and **B)** representative scatter plots of CCNA2 and cell cycle genes expression across the combined transcriptomic profiles of all subclusters of nTg and CCNA2-Tg mice. Bars represent mean ± s.e.m. Each point represents an individual value; the mean value is represented by the horizontal line. Error bars represent s.e.m.

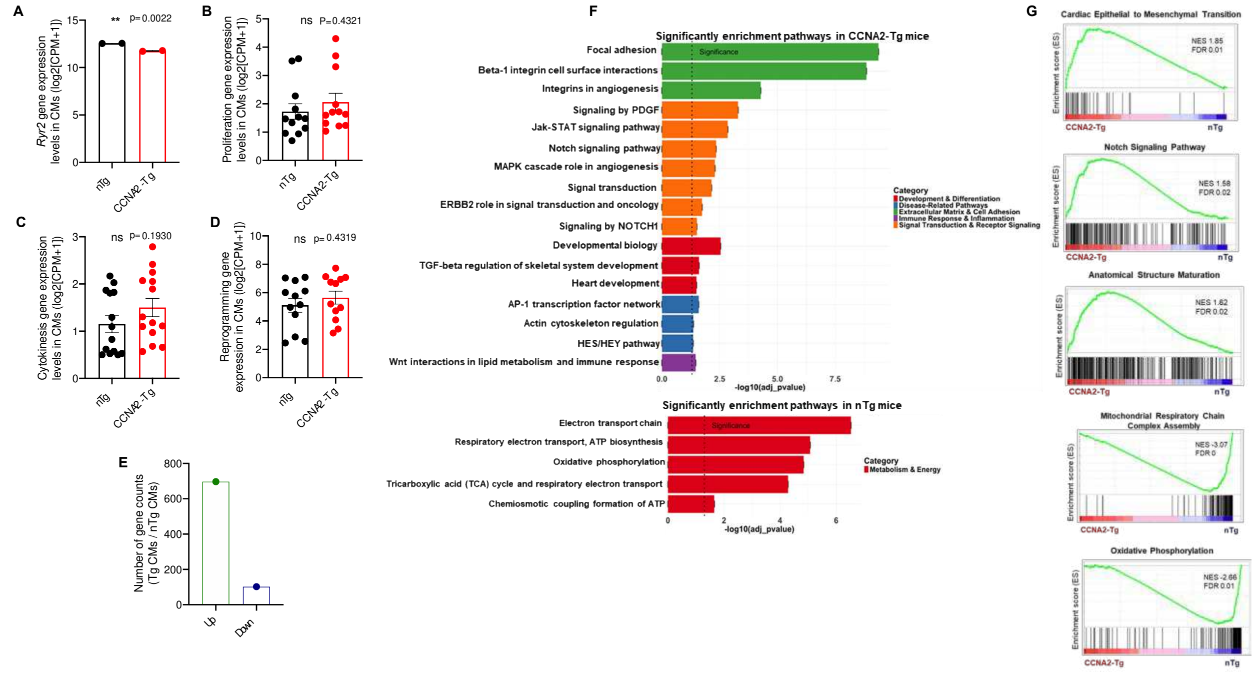

Figure S5. A) Representative scatter plots of cardiomyocyte maturation gene *Ryr2*, ***B)*** proliferation, **C)** cytokinesis, and **D)** reprogramming gene expression in nTg and CCNA2-Tg cardiomyocytes. Each dot represents an individual value, and bars indicate mean ± s.e.m. **E)** Number of differentially expressed genes (Up or Down) in adult CCNA2-Tg mouse cardiomyocytes as compared to adult nTg mouse cardiomyocytes. **F)** Significantly enriched pathways in adult CCNA2-Tg and nTg mouse cardiomyocytes (P-value adjusted <0.05), analyzed using BioPlanet. **G)** Gene set enrichment analysis (GSEA) in CCNA2-transgenic versus nTg cardiomyocytesshowing a significant downregulation of oxidative phosphorylation and mitochondrial respiratory chain complex assembly pathways in CCNA2-transgenic cardiomyocytes, may suggest a metabolic shift consistent with dedifferentiation. Conversely, pathways such as cardiac epithelial-to-mesenchymal transition (EMT) and Notch signaling were upregulated, indicating activation of developmental and reprogramming pathways that facilitate a more regenerative state.

Movie S1. The real-time, live-imaging movie of human adult cardiomyocytes (55-year-old male) as described in **Figure 1.**

Movie S2. Real-time, live-imaging movie of human adult cardiomyocytes (41-year-old female) as described in **Figure 1.**
